# Spectroscopic evidence of mixed angular momentum symmetry in non-centrosymmetric Ru$_7$B$_3$

**DOI:** 10.1038/s41598-021-99878-6

**Published:** 2021-10-26

**Authors:** Soumya Datta, Aastha Vasdev, Ranjani Ramachandran, Soumyadip Halder, Kapil Motla, Anshu Kataria, Arushi, Rajeswari Roy Chowdhury, Ravi Prakash Singh, Goutam Sheet

**Affiliations:** 1 Department of Physical Sciences, Indian Institute of Science Education and Research (IISER) Mohali, Sector 81, S. A. S. Nagar, PO 140306, Manauli, India; 2 Department of Physics, Indian Institute of Science Education and Research Bhopal, Bhopal, 462066 India

**Keywords:** Condensed-matter physics, Superconducting properties and materials

## Abstract

Superconducting crystals with a lack of inversion symmetry can potentially host unconventional pairing. However, till today, no direct conclusive experimental evidence of such unconventional order parameters in non-centrosymmetric superconductors has been reported. In this paper, through direct measurement of the superconducting energy gap by scanning tunnelling spectroscopy, we report the existence of both *s*-wave (singlet) and *p*-wave (triplet) pairing symmetries in non-centrosymmetric Ru$_7$B$_3$. Our temperature and magnetic field-dependent studies also indicate that the relative amplitudes of the singlet and triplet components change differently with temperature.

## Introduction

The BCS theory assumes that the attractive interaction that leads to superconductivity is isotropic in momentum space^1^. Consequently, the superconducting energy gaps of BCS superconductors show *s*-wave (orbital angular momentum, $l=0$) symmetry. In certain superconducting systems, the energy gap may become anisotropic in the momentum space, and show higher angular momentum symmetries like *p*-wave ($l=1$)^2–4^, *d*-wave ($l=2$)^5–7^ etc. In certain other systems, the existence of a mixed angular momentum symmetry, where symmetries represented by different *l* are mixed, has also been possible^8–11^. The physics of such non-*s*-wave superconductors are not understood within the BCS formalism. If the crystal structure of a superconductor lacks a center of inversion symmetry, parity is not a good quantum number. In such a system, an antisymmetric spin-orbit coupling (ASOC) can exist. ASOC can, in principle, remove the spin degeneracy of the Bloch states with the same *k* (crystal momentum) but opposite spins. In the presence of ASOC, the orbital angular momentum and spin angular momentum do not remain good quantum numbers any longer. Here, Pauli’s exclusion principle cannot restrict the symmetry of the Cooper pairs to be either purely even-parity singlet or odd-parity triplet. Therefore, a complex mixed angular momentum state becomes a possibility in a non-centrosymmetric superconductor^12^. The unconventionality associated with such complex angular momentum symmetry of the superconducting order parameters might make the non-centrosymmetric superconductors (NCS) exhibit unusual behavior in their electromagnetic properties compared to the purely *s*-wave superconductors. For example, they may display unusually high Pauli limiting fields^13^, helical vortex states^14^, and even topologically protected states^15^. Owing to these reasons, since the discovery of the first non-centrosymmetric superconductor CePt$_3$Si^13,16^, the study of such superconductors gained significant attention from the condensed matter physics community^17–19^.

Despite several theoretical predictions of the possibility of exotic superconducting phases in non-centrosymmetric superconductors as discussed above, there has been no clear spectroscopic evidence of unconventionality in such superconductors studied till date. In this paper, we report our ultra-low temperature scanning tunneling microscopy and spectroscopy results on a non-centrosymmetric superconductor Ru$_7$B$_3$. Ru$_7$B$_3$ belongs to the space group $P6_3mc$ and the cyclic crystallographic class $C_{6v}$^20^. Matthias *et* *al*. had first reported superconductivity in Ru$_7$B$_3$ in 1961^21^. However, owing to its low critical temperature, the system did not find much interest among the superconductivity community. Almost three decades later, the absence of the inversion symmetry in its crystal structure was highlighted by Morniroli et al.^22^. In various transport and thermodynamic measurements in the past^23–25^, it was seen that $\Delta C_e/(\gamma _nT_c)$ and 2$\Delta _0/(k_B T_c$) in Ru$_7$B$_3$ were approximately 1.4 and 3.3, respectively, indicating a weak-coupling superconducting state. These measurements also indirectly indicated that a predominant fully gapped *s*-wave order parameter could describe the superconducting state of Ru$_7$B$_3$ well. However, as we note, certain special features of the data presented in^23,24^ were ignored while claiming the absence of unconventional superconductivity in Ru$_7$B$_3$. The most intriguing among such special features was a kink in the field-dependent $\rho$-*T* data^24^, beyond which the superconducting transition curves split into two parts. The two parts exhibited significantly different sensitivity to the applied magnetic field and led to two dramatically different field scales for the upper critical fields ($H_{c2} \sim$ 1.1 T and 5 T respectively). In this context, Fang *et al.* discussed the possibility of a mixed angular momentum symmetry of the superconducting order parameter in Ru$_7$B$_3$. In addition, though it was ignored by the authors, a possible signature of unconventional pairing was also present in the specific heat data as presented in^23^. More recently, Cameron *et* *al*. performed small-angle neutron scattering^26^ on Ru$_7$B$_3$ and reported that the orientation of the vortex lattice in Ru$_7$B$_3$ strongly depends on the history of the applied magnetic field, thereby indicating the possibility of broken time-reversal symmetry in the order parameter. The superconducting order parameter is an energy-resolved quantity. Hence, a direct energy-resolved measurement technique, like scanning tunneling spectroscopy, is essential to understand its true nature. In RuB_2_, which is a centrosymmetric superconducting phase of the same elements Ruthenium and Boron, recently such tunneling spectroscopic data revealed unusual multi-band signatures in the order parameter^27^. Motivate by that, in order to probe the true order parameter symmetry of the non-centrosymmetric phase Ru$_7$B$_3$ also, we carried out a detailed temperature and magnetic field-dependent tunneling measurements on Ru$_7$B$_3$. The analysis of such data reveals the spectroscopic signatures of an order parameter with mixed angular momentum symmetry.

## Experimental methods

**Figure 1:**
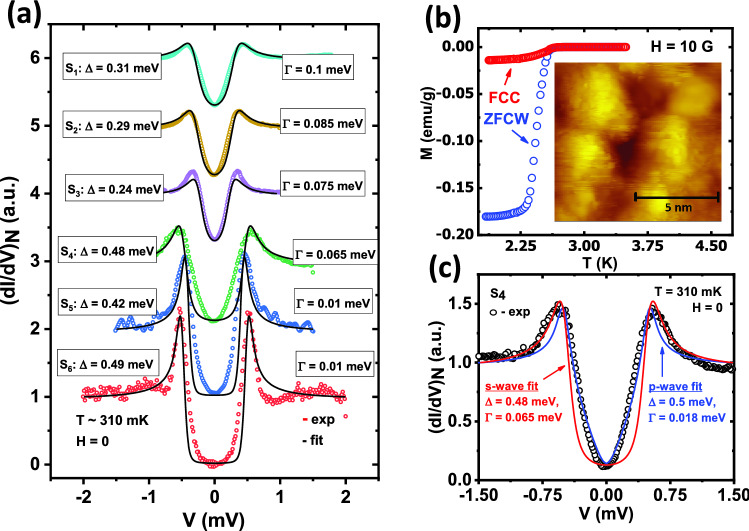
(**a**) Six representative tunneling spectra ($S_1$–$S_6$) plots (color points) along with corresponding numerically generated spectra under single gap *s*-wave model (black lines). The extracted fitting parameters $\Delta$ and $\Gamma$ are also shown for each spectrum. (**c**) Spectrum $S_4$ with both best single gap ‘*s*-wave’ and ‘*p*-wave’ fit along with the corresponding extracted parameters. The temperature (*T*) $\sim$ 310 mK for all spectra. (**b**) Bulk magnetization (*M*) data in both zero field cool warming (ZFCW) and field cool cooling (FCC) condition with 10 G magnetic field. *Inset*: STM topographic image of the sample.

The single crystals used for our measurements showed a bulk superconducting transition at 2.6 K (Fig. 1b). The scanning tunneling microscopy (STM) and spectroscopy (STS) experiments were performed in a Unisoku system with an RHK R9 controller, inside an ultra-high vacuum (UHV) cryostat at $\sim$ $10^{-10}$ mbar pressure. The lowest temperature down to which the measurements were performed was 300 mK. The STM is also equipped with a superconducting solenoid capable of producing a magnetic field up to 11 T. Since the single crystals could not be cleaved using the standard cleaving technique (optimized for layered materials only), we cleaned the surface by reversed sputtering for 30 min with Argon (Ar) ion in-situ inside an integrated UHV preparation chamber. Following that, we immediately transferred the sample to the scanning stage at low temperature. The Tungsten (W) tip, which was prepared outside by electrochemical etching, was also cleaned in UHV by bombarding it with a high-energy electron-beam. This process helped us probe the pristine surface of Ru$_7$B$_3$. In the inset of Fig. 1b, we show an STM topographic image showing the distinctly visible crystallites with average grain size $\sim$ 3 nm.

## Results and discussion

In Fig. 1a we show six representative tunneling spectra ($S_1$–$S_6$) captured at randomly chosen points on the surface of Ru$_7$B$_3$ at $\sim$ 310 mK. A visual inspection reveals that, the spectra can be distinctively divided into two categories based on their overall shapes. The first type ($S_1$, $S_2$, and $S_3$) shows coherence peaks around ± 0.30 mV and shallow ‘V’-shaped dip in between the peaks. The spectra ($S_4$, $S_5$, and $S_6$) of the second type show coherence peaks at ± 0.47 mV, and they have a higher curvature and depth (*dI*/*dV* $\sim$ 0 at *V*=0) below that. We have also analyzed these spectra within a single band ‘*s*-wave’ model^1^ using Dyne’s formula^28^: $N_s(E) \propto Re\left( \frac{(E-i\Gamma )}{\sqrt{(E-i\Gamma )^2-\Delta ^2}}\right)$. The tunnelling current is given by $I(V) \propto \int _{-\infty }^{+\infty } N_s(E)N_n(E-eV)[f(E)-f(E-eV)]dE$. Here, $N_s(E)$ and $N_n(E)$ are the density of states (DOS) of the superconducting sample and the normal metallic tip, respectively and *f*(*E*) is the Fermi-Dirac distribution function. $\Gamma$ is the Dyne’s parameter that takes care of the broadening of DOS. The theoretical plots thus generated are shown as black lines on the experimental data points in Fig. 1a. The spectra $S_1$, $S_2$ and $S_3$ give $\Delta$ of the order of 0.31 meV, 0.29 meV, and 0.24 meV, respectively while spectra $S_4$, $S_5$, and $S_6$ provide the values 0.48 meV, 0.42 meV, and 0.49 meV respectively from the same analysis. To note, while the first group of spectra ($S_1$, $S_2$, and $S_3$) shows reasonably good fitting with the single gap ‘*s*-wave’ model (albeit with large $\Gamma$), the second group ($S_4$, $S_5$, and $S_6$) exhibits a significant departure from that.

The above-mentioned discrepancy between the experimental spectra and the spectra generated theoretically within a single-band ‘*s*-wave’ model, prompted us to consider other possible symmetries of the order parameter. To perform such an analysis, we modified Dyne’s equation by introducing a more general expression^29^ of $\Delta (\theta )$ than an isotropic $\Delta$. The modified Dyne’s equation reads as $N_s(E,\theta ) \propto Re\left( \frac{(E-i\Gamma )}{\sqrt{(E-i\Gamma )^2-(\Delta ' Cos(n\theta ))^2}}\right)$. Here, $\theta$ is the polar angle (w.r.t. (001)) and the integer *n* can be 0, 1 or 2 for *s*, *p,* and *d* wave symmetries, respectively. The expression for tunneling current is also modified to $I(V) \propto \int _{-\infty }^{+\infty }\int _{0}^{2\pi } N_s(E,\theta )N_n(E-eV)[f(E)-f(E-eV)]d\theta dE$. In Fig. 1c we show the experimental spectrum $S_4$ along with theoretical plots considering isotropic ‘*s*-wave’ $\Delta$ (red line) and anisotropic ‘*p*-wave’ $\Delta$ (blue line). It is clear that the spectrum, especially the ‘V’-shaped part of that between the coherence peaks, is better described by the ‘*p*-wave’ symmetry. It is also interesting to note that the extracted value of $\Delta$ (0.47 meV) for such fit does not differ much from the best ‘*s*-wave’ fit (0.48 meV).

Such ‘V’-shaped STS spectra are often seen for superconductors with possible unconventional symmetries and are well described by the Tanaka-Kashiwaya model^29,30^ we used here. For example, considering various possible symmetries under the framework of an “anisotropic *s*-wave’ model in MgB$_2$, Seneor *et al.*^31^ explained spectroscopic signatures, *e.g.*, very high coherence peaks, ‘V’-shaped valley, and also zero-biased coherence peak (ZBCP). In anisotropic Bi$_2$Sr$_2$CaCu$_2$O$_8$, Ichimura *et al.*^32^ reported two types of spectra, one with a ‘V’-shape and another with a flat bottom. The authors explained those with a model of mixed ‘$s + d$-wave’ symmetry where the former isotropic component is the dominant one. Millo *et al.*^33^ reported both ‘V’-shaped spectra and spectra with ZBCP in SmFeAsO$_{0.85}$ and adduced such shapes with the ‘*d*-wave’ order parameter. To note, it was also reported there that some of the STM spectra could also be fitted well within a pure ‘*s*-wave’ model but with significantly smaller $\Delta$ and relatively higher $\Gamma$^33^—a situation similar to our group I spectra. From our analysis, an order parameter with either ‘*p*-wave’ or ‘*d*-wave’ symmetry can reproduce a ‘V’-shaped spectra as mentioned in Group II. However, as reported by Cameron *et al.*^26^, the spontaneous magnetic field present in Ru$_7$B$_3$ breaks the time-reversal symmetry in the order parameter and supports the ‘*p*-wave’ gap as a more favorable possibility.

**Figure 2:**
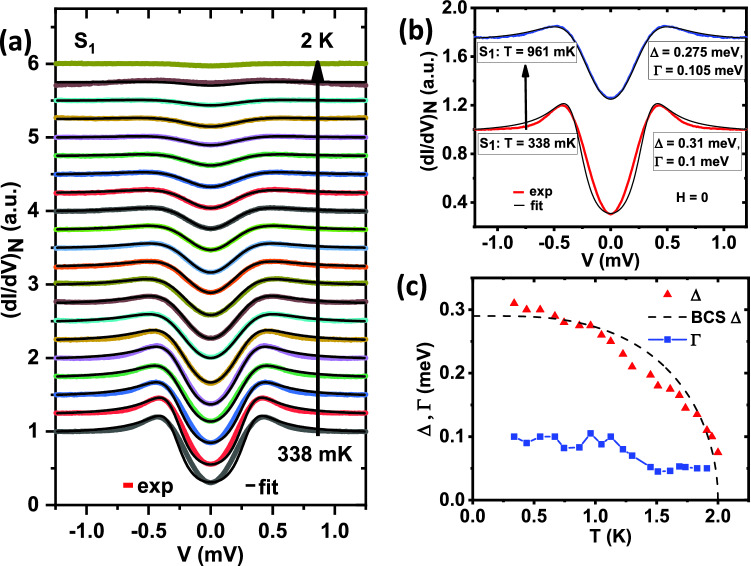
(**a**) Temperature (T) dependence of tunneling conductance spectra $S_1$ (color lines) with theoretical fits (black lines) in the absence of any magnetic field. (**b**) Spectra $S_1$ at 340 mK and also at 960 mK along with corresponding fitting parameters, where better fit at higher temperature is visible. (**c**) Evolution of $\Delta$ and $\Gamma$ with temperature, extracted from plot (a) along with an ideal BCS trend of $\Delta$ for comparison.

**Figure 3:**
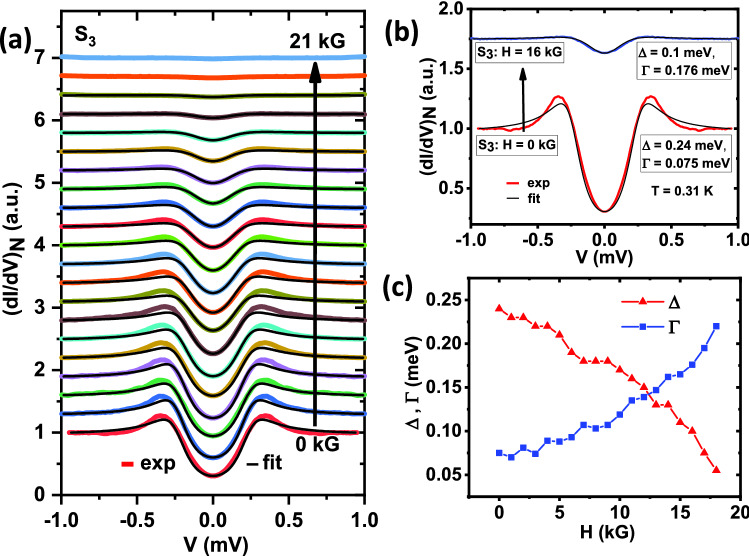
(**a**) Magnetic field (*H*) dependence of tunneling conductance spectra $S_3$ (color lines) with theoretical fits (black lines) all measured at T $\sim$ 310 mK. (**b**) Spectra $S_3$ in the environment of *H* = 0 and *H* = 16 kG field, along with corresponding fitting parameters. $H\parallel c$-axis of the crystal and a better fit at higher field is visible. (**c**) Evolution of $\Delta$ and $\Gamma$ with the magnetic field, extracted from plot (**a**).

Now we focus on the spectrum $S_1$, which belongs to group I and fits reasonably well with a single ‘*s*-wave’ gap (and with relatively large $\Gamma$). A closer inspection, however, reveals that there is a small discrepancy between the experimental data and the ‘*s*-wave’ model spectrum. We investigated the evolution of this discrepancy with temperature. Temperature dependence of $\Delta$ approximately followed the BCS prediction (Fig. 2c) with $\Delta _0$ = 0.3 meV. The broadening parameter $\Gamma$ did not change much within this range. The departure of the experimental spectrum from the *s*-wave model rapidly decreased with increasing temperature, and at around 750 mK, the discrepancy almost disappeared (Fig. 2a). To illustrate this effect clearly, we show two spectra, one at 338 mK and another at 961 mK, along with their theoretical (*s*-wave) fits in Fig. 2b. This observation indicates the possibility of a mixed angular momentum symmetry in the order parameter where the amplitudes corresponding to different *l* vary differently with temperature. This also explains why Fang *et* *al*. did not find any signature of unconventionality in their lower critical field ($H_{c1}$) studies^24^ down to 1.2 K, which is well above the temperature window where we could notice a deviation from the *s*-wave behaviour in our data. We have also performed magnetic field dependence of another spectrum ($S_3$) from the same group I (Fig. 3a). The key observation is that while the spectra deviate appreciably from ‘*s*-wave’ theoretical curves at low fields, above 10 kG the spectra resembled far more closely to the ‘*s*-wave’ predictions. For demonstrating this effect more clearly, we show the spectra and respective ‘*s*-wave’ fits both at zero field and at 16 kG, in Fig. 3b. The evolution of the extracted gap ($\Delta$) and broadening parameter ($\Gamma$) with the applied magnetic field are shown in Fig. 3c.

**Figure 4:**
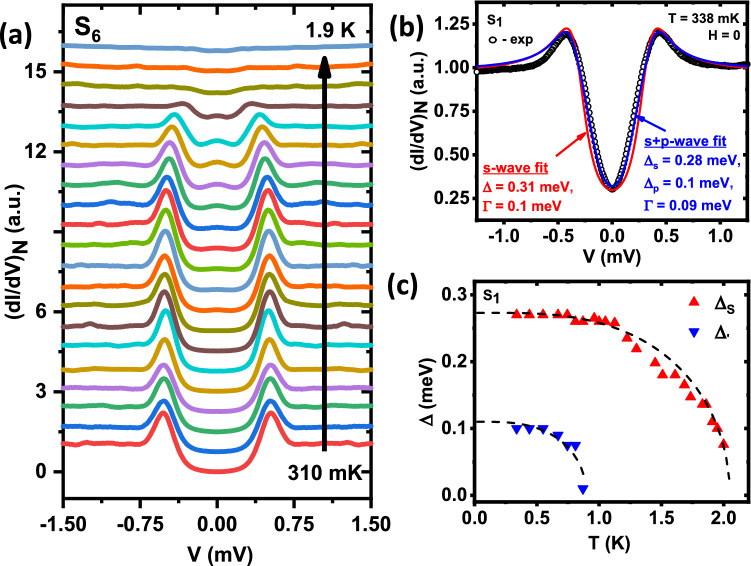
(**a**) Temperature (T) dependence of tunneling conductance spectra $S_6$ incorporating gradual appearance and disappearance of the peak-like feature. (**b**) Spectrum $S_1$ along with best pure *s*-wave (red line) and mixed $s+p$-wave (blue line) fits with corresponding extracted parameters $\Delta$ and $\Gamma$. (**c**) Evolution of $\Delta _s$ and $\Delta _p$ with temperature, extracted from the $s+p$-wave fits of spectra $S_1$. Ideal BCS trends of $\Delta$ are also shown for comparison.

In Fig. 4a, we demonstrated a detailed temperature dependent study of spectra $S_6$, which belong to group II. At $\sim$ 740 mK, a small zero-bias conductance peak (ZBCP) appeared. It became more and more pronounced up to $\sim$ 1370 mK and then started fading until it, along with other prominent spectral features, gradually disappeared at 1.9 K. To note, the emergence of such a ZBCP can be attributed to a ‘*p*-wave’ component in the order parameter where the interface normal and the lobe-direction of the ‘*p*-wave’ maintain an acute angle between them^34,35^. Since the surface of our Ru$_7$B$_3$ has crystallites with arbitrary orientations, and the tip is engaged randomly at different points, this condition can naturally be satisfied sometimes.

Based on the discussion above, if Ru$_7$B$_3$ has a mixed angular momentum symmetry in its order parameter, then we would expect the unconventional component to be present in group-I spectra too. To understand this aspect in detail, we used an ‘$s+p$-wave’ model to analyze the spectra $S_1$. In this model, the effective gap is given by $\Delta _{s+p}$ = $\Delta _s$ + $\Delta _p Cos\theta$. In Fig. 4b, we present the spectrum $S_1$ (black circles) at 338 mK with numerically generated spectra considering ‘$s+p$-wave’ symmetry (blue line). Pure ‘*s*-wave’ fit (red line) is also shown for comparison. It is clear that the mixed angular momentum symmetry provides a better description of the data. Temperature evolutions for the amplitudes of the two components $\Delta _s$ and $\Delta _p$ extracted from the above ‘$s+p$-wave’ fittings for the whole spectra $S_1$ are presented in Fig. 4c. It is not a surprise that, while the conventional $\Delta _s$ follows a smooth BCS like dependence up to 2 K, the smaller $\Delta _p$ sharply drops and goes beyond our measurement resolution at 0.9 K.

It should be noted here that the superconducting gap is the manifestation of a phase-coherent macroscopic condensate. Therefore, the measured $\Delta$ should ideally be unique irrespective of the measurement techniques. Since the system is metallic in nature^23,24^, any role of special surface states can be ruled out. The absence of multiple distinct pairs of coherence peaks in any spectra makes the possibility of a multi-band superconductivity or a proximity-induced superconductivity (PIS) in the bottom of the STM tip unlikely. In a different context, a similar variety of transport spectra is possible if some superconducting nanoislands are deposited and separated spatially from each other on an otherwise non-superconducting metal or insulator^36,37^. PIS is reported on such non-superconducting substances in the neighborhood of those superconducting islands, which eventually gets vanished while moving away from the islands. Nevertheless, in our case, the whole surface under the tip is of a pure superconducting Ru$_7$B$_3$ single-crystal, unlike such geometry, and we did not find an entirely gap-less flat spectrum anywhere on the surface. Also, as reported by Cherkez *et al.*^37^, spectra probed on the superconducting islands and away from the islands, both could be well fitted with the BCS model with just different sets of parameter values. However, in our case, a pure BCS model fails to explain the spectra, and an anisotropic component, whether relatively small ( for group I ) or large ( for group II ) is essential. In that way, we can safely exclude the possibility of any proximity effect in our measurements. The $T_c$ and the $H_c$ that we measure for all our spectra match well irrespective of whether they fall under Group I or II. Therefore, it can be concluded that all the spectra falling under two distinct groups differ from each other based on which component of the order parameter contributes predominantly for a particular crystallite that the measurement is performed on.

## Conclusion

In summary, we performed scanning tunneling spectroscopy on single-crystal Ru$_7$B$_3$ and recorded several spectra, which can be broadly categorized into two groups, group-I and II. Group-I consists of spectra that are shallow in shape. They show overall good agreement with single gap ‘*s*-wave’ symmetry except at very low temperatures where they deviate from such a pure *s*-wave model. Smaller superconducting gaps and larger broadening parameters are characteristics of these spectra. Group-II spectra are broader in shape and show sharper coherence peaks. They significantly deviate from the predictions of the ‘*s*-wave’ model, but a theoretical model with ‘*p*-wave’ symmetry shows a better agreement. The superconducting gaps are larger, and the broadening parameters are very small for such spectra compared to those belonging to the first group. The temperature dependences of the spectra belonging to both groups indicate the presence of a mixed ‘$s+p$-wave’ symmetry in the order parameter where the two components have different temperature dependence.
